# COVID-19 Outbreak and Financial Performance of Chinese Listed Firms: Evidence From Corporate Culture and Corporate Social Responsibility

**DOI:** 10.3389/fpubh.2021.710743

**Published:** 2021-09-17

**Authors:** Yunpeng Sun, Ying Li

**Affiliations:** School of Economics, Tianjin University of Commerce, Tianjin, China

**Keywords:** coronavirus, financial performance, Chinese listed companies, corporate culture, corporate social responsibility (CSR)

## Abstract

This research described Chinese listed firms' COVID-19 Outbreak and financial performance using corporate culture (CC) and corporate social responsibility (CSR) evidence. The epidemic's impact on Chinese companies' profits was much less than the impact on their sales growth rates. Although the COVID-19 has had a more significant negative impact on the financial performance of Chinese listed companies in sectors that are more severely impacted, such as travel and entertainment, we believe that the financial performance of the medical industry has improved as a result of the outbreak. Meanwhile, Chinese listed companies in high-risk areas experience more significant financial losses during the epidemic, and the Hubei impact is hefty weight. Corporate social responsibility moderated the inverse relationship between this epidemic and Chinese firms' economic success. This research enhances the current literature on the effects of the COVID-19 on financial success and practical, realistic, and theoretical consequences in companies worldwide.

## Introduction

The first cases of the Covid-19 outbreak were recorded in Wuhan, China, on December 31, 2019, marking a key turning point in the outbreak's expansion to other Chinese provinces (1). As a result of the situation, the Chinese government agreed to close Wuhan on January 23, 2020, restricting outbound and inbound travel. The Chinese economy slowed due to the extreme lockout, as shown by a drop in manufacturing outputs and retailing income and a rise in unemployment. According to Bloomberg, Chinese industrial outputs plummeted by 13.5% in January and February 2020, whereas the retailing sector's revenue declined by 20.5%, in February 2020, the unemployment rate reached 6.2%. According to the IMF, Chinese economy will cool by around 0.4% points. The financial results of Chinese listed companies are likely to suffer in the first quarter of 2020 due to this epidemic. However, the effects of the COVID-19 on firm financial results have not been investigated in Chinese literature in particular; even though assessing the outbreak's effects is crucial for firms to decide their appropriate market plans. Furthermore, there is a paucity of literature on the effects of infectious diseases on firm financial performance since the bulk of the literature on the subject has concentrated on the effects of such diseases on stock market performance (2, 3). Scholars discovered that dangerously contagious infections harmed consumer sentiment, affecting investment decisions (4). Consequently, when the dangerously infectious epidemic erupted, equity markets displayed a greater degree of uncertainty formed by erratic investor behavior (5). Notably, the stock market can overreact to certain diseases due to herding behaviors and panic spread, and stock market performance may not accurately represent firm financial efficiency. As a result, without assessing the economic effects of such illnesses, businesses will be unable to adequately evaluate their plans for dealing with and recovering from the health crisis. This analysis aims to add to the data on the effects of the Covid-19 outbreak on Chinese firms' financial. The epidemic affected the financial performance of Chinese publicly traded companies as measured by the sales growth rate, return on assets (ROAs), return on equity (ROE), and asset turnover (ATO) (6, 7). After the epidemic began in China at the end of the fourth quarter of 2019 and deepened in the first quarter of 2020, the quarterly data more reliably represents the effect of the outbreak on firm financial results and China had successfully contained the epidemic, reducing the effects of the outbreak on businesses. Following that, China effectively handled the epidemic and, unlike other nations, did not suffer a second phase of the health crisis due to the government's tight measures and Chinese companies recovered from the recession and did well in other areas. Since this analysis uses panel results, the generalized least squares (GLS) with cross-section weights and the white cross-section covariance approach are used to deal with cross-section heteroscedasticity and residual cross-section dependency (8). In general, the Covid-19 outbreak harmed Chinese listed firms' financial results in the first quarter of 2020, as it depressed the sales growth rate, ROA, ROE, and ATO of the companies analyzed in that quarter. Furthermore, the outbreak's negative effects on Chinese firms' sales growth rate were most severe this quarter, with revenue falling by 40%. However, the epidemic had only a minor impact on Chinese listed companies' ROA, ROE, and ATO, only 0.3, 0.7, and 5.2%, respectively. The outbreak's impact on the financial performance of Chinese publicly traded companies varied by industry and businesses with various working capital management policies and capital structures. Furthermore, corporate culture (CC) and corporate social responsibility (CSR) mitigate the detrimental impact of the epidemic on Chinese firms' financial results. The rest of the analysis divides into four parts after the introduction. The research history and current results on the effects of dangerously infectious diseases on firm efficiency clarify in the literature review section. The methodology segment describes the data collection process, sampling procedure, test design, hypotheses created, and research models developed. We discussed the details of the data collection in the fourth segment, the research's accomplishments and shortcomings outlined, and recommendations to the research's related stakeholders proposed.

## Literature Review

### Firm Performance

There are different theoretical perspectives of firm performance. The shareholder theory focuses on financial performance measures to examine how healthy firms maximize their profit to satisfy shareholder's needs. Differently, the stakeholder theory considers the satisfaction of all stakeholders as an indicator of firm performance (9). The balanced scorecard measures athletic performance by four aspects—financial, customer, internal process, and learning and growth (10). According to the balanced scorecard perspective, firms with excellent knowledge, and growth performance can obtain a better internal process (11). The firms are profitable, they can offer more incentives to their employees, leading to better employee satisfaction and retention (12). Firms' financial performance refers to their effectiveness and efficiency in utilizing their assets and capital to generate revenue and profit. It measures several profitability measures (ROA, ROE, ROCE) and turnover ratios and profitability ratios measure the ability of firms to generate profit (13).

In contrast, turnover ratios measure the efficiency of firms in utilizing their assets and capital in generating revenue (14) and a turnover ratio as indicators of agency costs (15). If agency conflicts are high, the management tends to underperform (16), leading to a low ATO ratio as assets utilizes efficiently (17). On the other hand, firm performance measures by the market performance of firms' shares proxied by Tobin's Q (18). However, firms' market performance may be subjective to investor sentiment and other irrational behaviors (19). Therefore, to examine firms' performance of firms, accounting based measures are preferred since they reflect firms' intrinsic performance without influences of external factors.

### Internal Determinants of Firm Performance

The literature indicates several determinants of firm performance and from the agency perspective, agency conflicts influence athletic performance as the management is self-interest-oriented (20). Hence, developing effective corporate governance mechanisms minimized agency conflicts to improve firm performance and empirically, the robust corporate governance mechanisms are not enough to justify firms' performance (21–23). Instead, other factors such as the effectiveness of business strategies and management capabilities determine how firms perform (24, 25). Besides agency conflicts, working capital strategies adopted by firms also influence their financial performance (26) and customer satisfaction increases. By contrast, the minimization of current assets, called the aggressive working capital management, minimizes costs incurred by firms since inventories are only replenished when demands emerge. Consequently, firms' profitability increases, this strategy is risky as firms may not respond effectively to customers' demands and may have to borrow additional funds to accommodate their short-term liabilities. Empirically, a large number of research studies support the trade-off between liquidity and profit as well as the negative impacts of the conservative working capital management on firm performance (27). On the other hand, the management of long-term debts is also critical since it influences firms' profitability (28) and firms have two sources of funds—debts and equity. The ratio between debt and equity determines firms' capital structure. According to the financial leverage is a “double-edge sword” since it can provide both benefits and threats to firms. When firms increase their debts, they can gain tax shield benefit since interest expenses are tax deductible. Furthermore, with the issuance of debts, firms can avoid the dilution of their current shareholders' benefits and the production perspective, firm size is also a determinant of athletic performance. Thus, firms can improve their profitability compared to others according to Josefy et al. (29), a giant size requires extensive capital investment and a highly structured organization.

### Corporate Culture and Firm Performance

Corporate culture is a collection of unique values and codes of conduct shared by a company's people. Corporate culture is the soul and core of an enterprise and it is a critical factor that determines the firm performance and sustainable development. Arogyaswamy and Byles (30) stated that when the CC had consistent values or beliefs with the corporate strategy, it positively affected its performance. Denison (31) argued that CC affected a company's financial performance: business strategy, employees' work status, innovation activities, and financial information. Argued that CC indirectly affected firm performance through the moderating role of market performance and this strength in a dynamic market.

Similarly, Flamholtz and Kannan-Narasimhan (32) also advocated that CC indirectly correlated with firms' financial performance. Molenaar et al. (33) stated the safety culture of firm had a positive correlation with the construction performance. Huhtala et al. (34) found that the ethical culture has a significant positive effect on managers' professional performance and work participation behavior. Kaptein (35) argued that the CC could promote the employees' awareness and behavior. Stated that if a company ignored the function of the CC in governance, it would cause a huge damage to the firm performance. Argued that companies with an innovative culture could promote their performance through the role of the managers with their high-level emotional intelligence. Vigolo et al. (36) found that the service-oriented CC could help to increase employee motivation and satisfaction, and thereby improved firm performance. Pinho et al. (37) argued that the different types of culture had different effects on the firm performance and Zhao et al. (38) found that CC improvements are negatively linked with firm value, and positively related to innovation outputs. In particular, CC can better affect employees' job satisfaction and strengthen their organizational commitments.

### Corporate Social Responsibility and Firm Performance

Explained emerged in the 1950s by four distinct theoretical perspectives: instrumental, political, integrated, and ethical. The first theoretical perspective considers CSR a vehicle for firms to achieve their profit targets and maximize shareholders' wealth. The second advocates that CSR is needed to realize their political power and present their corporate citizenship (39–41). The third theoretical perspective argues that CSR is essential to firms since they need to discharge their accountability to different resource holders through such activities (9). Thus, firms need to integrate the demands of other stakeholders into their business strategies and performance (42). Despite their differences in explaining the role of CSR, these theories share the standard definition of CSR and environmental activities of firms that ensure their compliance with legal and ethical frameworks as well as the “self-enlightening” perspective (40). This linkage commonly agrees upon the link between CSR and firms' performance (43). The integrative theory indicates that CSR brings rewards to firms because it enables firms to satisfy the demands of different stakeholders (44) to prevent reputational risk facing firms (45), to increase employee engagement and long-run stock return (46). Differently, CSR effects on firm performance may also be neutral because its benefits offset the additional costs added to firms (47). The neutral effects of CSR on firms' market performance are also justified by the impacts of external factors on share prices, as stated by behavioral finance (48).

### Dangerously Contagious Diseases and Firm Performance

Dangerously contagious diseases refer to diseases, which are highly contagious among people and require extensive healthcare resources to prevent and cure. Examples of such conditions are Spain Flu, SARS, MERS, Ebola, and COVID-19 (49). Consequently, unemployment, income decreases, consumer expenditure declines, and leading to macroeconomic headwinds (1). The literature on the impacts of such diseases on firms' financial performance is scarce since most of the existing research studies focus on examining the effects of such conditions on the stock market (2–4).

However, the heterogeneity in impacts of such diseases on the stock market found that investors of this sector expect vaccine development to maximize returns of pharmaceutical stocks in the future (4). The COVID-19 outbreak negatively influenced most stock markets in the outbreak severely infected countries (2, 3). Jung et al. (50) examined the impacts of the MERS outbreak on customer expenditures in Korea. They found that total spending reduced significantly due to this outbreak and still, the heterogeneity existed across categories Secinaro et al. (6) examined how influenced the performance of medium companies in the travel and leisure sector in Europe. This research employed the content analysis of annual reports to compare three companies' financial performance and position in 2002 and 2003 (SARS). Aifuwa et al. (7) investigated the impacts of COVID-19 on Nigerian firms' financial and non-financial performance. Hassan et al. (51) revealed that since the emergence of the Covid-19 outbreak, firms mainly were concerned with the collapse of demand, the increase of uncertainty, and the disruption of their supply chain, the reduction of capacity, closures, and employee welfare. Furthermore, many firms could also foresee business opportunities in new and disrupted markets caused by the spread of this disease and the firms, which had experience with SARS, presented their positive expectations about their ability to respond to this outbreak.

## Research Methodology

### Data Collection Procedure

This research adopts the stratified sampling method (52) to select Chinese listed firms as samples and it divides the total population into different segments and then randomly selects selections from each piece. This method enables random samples selected across various components and in this research, the total population of Chinese listed firms divides into 16 different industries. The final sample size is 126 listed firms from both Shanghai and Shenzhen stock markets and these firms are classified into 16 sectors, as shown in Table 1. For each sample, the quarterly financial data gather from Wind Database from the second quarter of 2019 to the second quarter of 2020. Among quarters, Chinese firms were not affected COVID-19 outbreak Q2, Q3, and Q4 of 2019, whereas this outbreak influenced them in Q1 and Q2 of 2020.

**Table 1:** The general profile of samples by industries.

**No**	**Industry type**	**Number of companies**	**Percentage (%)**
1	Airlines	10	8.06
2	Tourism	12	9.68
3	Retailers	10	8.06
4	Healthcare	6	4.84
5	Basic materials	16	12.90
6	Automobile	1	0.81
7	Energy	5	4.03
8	Industrials	10	8.06
9	Food processing and agriculture products	9	7.26
10	Entertainment	1	0.81
11	Construction	8	6.45
12	Beverage	7	5.65
13	Textile	10	8.06
14	Paper manufacturing	5	4.03
15	Technology	9	7.26
16	Real Estate	7	4.03

### Hypothesis Development

The COVID-19 outbreak, after its emergence in December 2019 in Wuhan, spread China nationwide rapidly. The COVID-19 attack reduced the financial performance of Chinese listed firms in the first quarter of 2020.

**H**_**1**_**:** The COVID-19 outbreak reduced the financial performance of Chinese listed firms.

According to Donadelli et al. (4), a dangerously contagious disease may negatively affect performance. Furthermore, stated that in the context of the contagiousness of the COVID-19 outbreak, demands for commodities and manufactured products declined, whereas requests for medical supplies and food products soared and the impacts of this outbreak on the financial performance of Chinese listed firms in different industries might not be convergent.

**H**_**2**_: There is heterogeneity in impacts of the COVID-19 outbreak on the financial performance of Chinese listed firms in different industries.

Under this scenario, companies in different regions shut down their production line, negatively affecting their supply chain. For the demand side, the household's consumption demand suppress by the COVID-19 outbreak (1). Thus, the impacts of this outbreak on the financial performance of Chinese listed firms in severe epidemic regions might be more potent.

**H**_**3**_: The impacts of COVID-19 outbreak on the financial performance of Chinese listed firms in painful areas is substantial.

It is a psychological contract among people that enables them to have mutual recognition and understanding about the relationship and interaction between employees, managerial positions, and the entire organization in general. The CC can foster people to innovate and figure out the best way to protect themselves from this outbreak under the supports of their firms as well as to maximize firms' performance. Thus, the CC is hypothesized to have moderating effects on the relationship between the COVID-19 outbreak and Chinese listed firms' financial performance.

**H**_**4**_**:** The corporate culture has moderated impacts of the COVID-19 outbreak on the financial performance of Chinese listed companies.

The stakeholder theory (9) and integrated theories of CSR (42) suggest that the investment in CSR enables firms to satisfy better stakeholders' demands, leading to better employee engagement, a more sustainable supply chain, and the higher reputational position. The CSR hypothesizes is to have moderating effects on the impacts of the COVID-19 outbreak on the financial performance of Chinese listed companies.

**H**_**5**_**:** Corporate social responsibility has moderate effects on the negative impact of COVID-19 on the financial performance of Chinese listed companies.

China differentiates itself from other countries because of its political-economic model and it aims to develop the socialist market economy, in which the state's firms (SOEs) play the critical role as this economy's backbone (53). Therefore, when this outbreak spreads, SOE scan easier access to government supports to deal with this crisis than non-state own firms. The adverse effects of this outbreak on SOE's financial performance, hence, may be lessened.

**H**_**6**_**:** Chinese SOEs experience fewer adverse effects of the COVID-19 outbreak on their financial performance than non-state own firms.

### Research Models

This research develops several research models that capture the impacts of the Covid-19 outbreak on Chinese listed firms' financial performance.


(1) $$\begin{array}{l} REG/ROA/ROE/ATO=\alpha +\beta_{1}COVID19+\beta_{2}FCC+\beta_{3}FCSR\\ \;+\beta_{4}Liquidity+\beta_{5}Efficiency+\beta_{6}Leverage\\ \;+\beta_{7}SO+\beta_{8}Size+\beta_{9}Industry+\varepsilon \end{array}$$

In which, Chinese firms' financial performance is proxy by the revenue growth rate (REG), ROA, ROE, and ATO. Revenue growth rate measures by the logarithmic value of a Chinese firm's revenue generated in the quarter_*t*_ scaled by its revenue generated in the quarter_t−1_. Return on asset measures by a Chinese firm's net income generated at the end of each quarter scaled by its corresponding total assets. The measure of ROE is the ratio between a Chinese firm's net payment at the end of each quarter and its total related equity. Asset turnover measures a Chinese firm's revenue generated at the end of each quarter scaled by its corresponding total assets. While the REG is a simple measure of Chinese listed firms' ability to generate revenue, ATO measures firms' capabilities in generating income compared to their total assets (13). Asset turnover, thus, is also a proxy of agency costs (17). Differently, ROA and ROE are two measures of profitability of Chinese listed firms' payment referring to the CSR factor, and CC, referring to the CC, is constructed based on the Principal Component Analysis (PCA) method. Key variables of CSR and CC retain when their Eigenvalues are higher than 1. Subsequently, the CSR and CC indexes are constructed based on the identified weight of each variable distributed to each sample scaled by the gap between the max and min weight of all models. The name of each raw variable used to measure CSR and CC their measurement provided. On the other hand, the effect of the Covid-19 outbreak may vary across industries, state ownership, and regions classified by geographic locations, and the seriousness of the Covid-19 research models developed to test for the heterogeneity of this outbreak's effect. The effect of COVID-19 would be more significant for Chinese listed firms in the medicine/pharmaceutical industry.


(2) $$\begin{array}{l} REG/ROA/ROE/ATO=\alpha +\beta_{1}COVID-19\\ \;+\delta_{1}COVID19^{*}Medicine\\ \;+\delta_{2}COVID-19^{*}ATE+\beta_{2}CC\\ \;+\beta_{3}CSR+\beta_{4}Liquidity+\beta_{5}Efficiency\\ \;+\beta_{6}Leverage+\beta_{7}SO+\beta_{8}Size\\ \;+\beta_{9}Industry+\varepsilon \end{array}$$

While β_1_ measures the average effect of COVID-19 outbreak, (β_1_ + δ_1_) measures the specific impact of COVID-19 on Chinese listed firms in the medicine-related industry. (β_1_ + δ_2_) measures the particular impact of the COVID-19 outbreak on Chinese listed firms in related Airlines, Tourism, and Entertainment industry, named as ATE. The impact of this outbreak may vary across regions, including the dangerously unstable region and the dangerously high risky region. Therefore, several research models develop to capture the heterogeneity of such effects across regions. For the dangerously hazardous parts, research models design as follows:


(3) $$\begin{array}{l} REG/ROA/ROE/ATO=\alpha +\beta_{1}COVID-19\\ \;+\delta COVID19^{*}DSR+\beta_{2}DSR+\beta_{3}CC\\ \;+\beta_{4}CSR+\beta_{5}Liquidity+\beta_{6}Efficiency\\ \;+\beta_{7}Leverage+\beta_{8}SO+\beta_{9}size\\ \;+\beta_{10}industry+\varepsilon \end{array}$$

DSR represents the dangerously severe regions affected by the COVID-19 outbreak, measured by the number of the confirmed COVID-19 cases/provincial (city) population; if this ratio equals 1, the number of the established cases/regional (city) population is more than the median of the country. Thus, these regions recognize as seriously affected by the COVID-19 outbreak. For the dangerously unstable areas, research models developed as:


(4) $$\begin{array}{l} REG/ROA/ROE/ATO=\alpha +\beta_{1}COVID19+\delta \;COVID19^{*}DHR\\ \;+\beta_{2}DHR+\beta_{3}CC+\beta_{4}CSR\\ \;+\beta_{5}Liquidity+\beta_{6}Efficiency\\ \;+\beta_{7}Leverage+\beta_{8}SO+\beta_{9}Size+\varepsilon \end{array}$$

Dangerously hazardous regions (DHR) represents the high-risk regions in the COVID-19 outbreak and measures the number of COVID-19 death cases/ provincial (city) population = 1. It indicates the number of confirmed cases/local (city) population is more than the median; these regions are high-risk of the COVID-19, several regression models developed.


(5) $$\begin{array}{l} REG/ROA/ROE/ATO=\alpha +\beta_{1}COVID-19\\ \;+\delta \;COVID-19^{*}EAST+\beta_{2}CSR\\ \;+\beta_{3}Liquidity+\beta_{4}Efficiency\\ \;+\beta_{5}Leverage+\beta_{6}SO+\beta_{7}Size+\varepsilon \end{array}$$

(6) $$\begin{array}{l} REG/ROA/ROE/ATO=\alpha +\beta_{1}COVID-19\\ \;+\delta \;COVID-19^{*}CENTRAL+\beta_{2}CSR\\ \;+\beta_{3}Liquidity+\beta_{4}Efficiency\\ \;+\beta_{5}Leverage+\beta_{6}SO+\beta_{7}Size+\varepsilon \end{array}$$

(7) $$\begin{array}{l} REG/ROA/ROE/ATO=\alpha +\beta_{1}COVID-19\\ \;+\delta \;COVID-19^{*}West+\beta_{2}CSR\\ \;+\beta_{3}Liquidity+\beta_{4}Efficiency\\ \;+\beta_{5}Leverage+\beta_{6}SO+\beta_{7}Size+\varepsilon \end{array}$$

We developed four research models to explore this outbreak's effect across the CC and CSR of Chinese state-own listed companies as following:


(8) $$\begin{array}{l} REG/ROA/ROE/ATO=\alpha +\beta_{1}COVID-19\\ \;+\delta COVID-19^{*}DSO+\beta_{2}CC\\ \;+\beta_{3}COVID-19^{*}DSO^{*}CC\\ \;+\beta_{4}CSR+\beta_{5}COVID-19^{*}DSO^{*}CSR\\ \;+\beta_{6}Liquidity+\beta_{7}CCC+\beta_{8}Leverage\\ \;+\beta_{9}Size+\varepsilon \end{array}$$

### Data Analysis Techniques

As mentioned in section Hypothesis Development, six hypotheses were developed and tested through the conduction of the cross-sectional regressions, which refer to regressions for the panel data. These regressions consider the problems of the panel data heteroscedasticity, autocorrelation, and effects of either cross-section. To determine which type of cross-sectional regressions is the best suitable for panel data gathered, firstly, the Ordinary Least Squares (OLS) perform for the developed models (54). Then, the cross-section dependence test performs to figure out whether the cross-section heteroscedasticity exists. The residual tests are also committed to figuring out whether autocorrelation of residuals appears. The GLS has performed the cross-sectional weights in the contemporaneous heteroscedasticity.

## Research Findings and Discussions

### Descriptive Statistics

Table 2 provides key statistics of the variables of 126 firms involved in this research. Among four past quarters, the quarter ended on March 31, 2020, witnessed the spread of the Covid-19 outbreak, whereas the three remaining quarters did not. Outbreak measured by a binary variable with one coded for the period with this outbreak and 0 for otherwise, its mean is 0.25, implying that this outbreak just existed in one over four studied quarters. Regarding the financial performance and position of Chinese firms, in four quarters (Q2, 2019 to Q1, 2020), the REG of Chinese listed firms was negative on average (−0.151). However, their ROA, ROE, and ATO were positive with 0.005, 0.011, and 0.017, respectively. Despite their average negative REGs, Chinese listed firms maintained positive profitability and ATO ratios in the past four quarters. The positive average ROA, ROE, and ATO reflect the possibility of positive gains from the COVID-19 outbreak in some industries in China. These gains offset the losses of others, leading to the slightly positive average profitability and ATO ratios of Chinese firms in the studied quarters. Chinese listed firms' CC and CSR indexes were more than 0.67 and 0.58, respectively, with a moderate standard deviation. Chinese listed firms had relatively good CC and CSR, with average CC and CSR differences among firms.

**Table 2:** Descriptive statistics of variables.

	**Mean**	**Median**	**Maximum**	**Minimum**	**Std. Dev.**	**Probability**
REG	−0.151	−0.033	3.045	−4.849	0.642	0
ROA	0.005	0.007	0.097	−0.529	0.038	0
ROE	0.011	0.016	0.263	−1.09	0.085	0
ATO	0.17	0.136	0.645	−0.003	0.123	0
FCC	0.678	0.65	0.872	0.367	0.327	0
FCSR	0.589	0.557	0.883	0.252	0.213	0
Liquidity	1.735	1.253	10.903	0.222	1.557	0
Efficiency	631.324	193.925	42,145.13	−68,613.55	4,432.74	0
LEV	0.148	0.089	0.731	0.000013	0.177	0
STO	17.294	0.000002	82.3	0.000006	24.601	0
SIZE	9.814	9.634	14.545	4.435	1.94	0.002
COVID-19	0.25	0.000098	1	0.076	0.434	0

### Correlation Analysis Results

Table 3 provides the bivariate associations between variables. Except for the strong positive correlation between ROA and ROE (0.95), the moderate correlation between REG and CCC (−0.544), the average correlation between size and capital structure is (0.596), and the moderate correlation between CC and CSR figure out.

**Table 3:** Correlation analysis results all variables.

	**REG**	**ROA**	**ROE**	**ATO**	**COVID-19**	**LIQ**	**Eff**	**LEV**	**STO**	**SIZE**	**CC**	**CSR**
REG	1											
ROA	0.186^***^	1										
ROE	0.151^***^	0.959^***^	1									
ATO	0.314^***^	0.130^***^	0.121^***^	1								
COVID-19	−0.380^***^	−0.006^*^	0.004^*^	−0.215^***^	1							
LIQ	−0.083^**^	0.061^**^	0.022^*^	−0.160^***^	0.017^*^	1						
Eff	−0.544^***^	−0.033^**^	−0.017^**^	−0.196^***^	0.133^***^	0.193^***^	1					
LEV	−0.011^*^	0.037^*^	0.085^*^	−0.178^***^	0.011^*^	−0.300^***^	0.004^*^	1				
STO	−0.006^*^	0.061^*^	0.082^*^	0.081^*^	−0.003^*^	−0.146^***^	−0.052^*^	0.187^***^	1			
SIZE	0.042^*^	0.127^***^	0.173^***^	0.085^*^	0.005^*^	−0.382^***^	−0.063^*^	0.596^***^	0.366^***^	1		
CC	0.068^**^	0.054^**^	0.053^**^	0.061^**^	−0.052^**^	0.001^*^	0.049^*^	0.0002^*^	−0.034^*^	0.004^*^	1	
CSR	0.089^**^	0.072^**^	0.075^**^	0.081^**^	−0.078^**^	0.019^*^	0.066^**^	0.011^*^	−0.024^*^	0.352^***^	0.532^***^	1

*** *is significance level at 0.01,*

** *is 0.05 significance level;*

* *is 0.1 significance level*.

However, since ROA and ROE would not be involved in the same regressions, the high degree of association between them does not matter to the regression quality. The moderate correlations found in this research would not highly likely create the multi-co linearity of regressions.

### Regression Analysis

#### Effects of COVID-19 Outbreak on the Financial Performance of Chinese Listed Companies

Table 4 provides the GLS outcomes, using the white cross-section covariance method and Cross-section weights. This regression method adopts since the cross-section's heteroscedasticity has been detected from the Cross-section dependence test (55). In all models, coefficients of the COVID-19 outbreak variable are significant at 0.01 levels so that this outbreak can predict Chinese firms' financial performance proxy variation by four mentioned measures. Coefficients of the COVID-19 outbreak variable are −0.3994 in model-1, −0.003 in model-2, −0.008 in model-3, and −0.052 in model-4.

**Table 4:** Generalized Linear Regression (GLR).

**Dependent variable**	**REG**	**ROA**	**ROE**	**ATO**
C	0.007002	−0.021072	−0.057966	0.104588
COVID-19	−0.399433^***^	−0.003005^***^	−0.007744^***^	−0.052164^***^
CC	0.004927^***^	0.003433^***^	0.002093^***^	0.001077^***^
CSR	0.002372^***^	0.003412^***^	0.002109^***^	0.015577^***^
Liquidity	−0.00156	0.002067^*^	0.001078^*^	−0.000905
CCC	0.000001^*^	0.00000101	0.000001	0.000025^*^
Capital structure	−0.004837	−0.002184^*^	−0.003275^*^	−0.004159^***^
State ownership	0.000585^*^	0.00000035	0.0000002	0.000257
Size	0.001328	0.002298^*^	0.006062^*^	0.001277
*F*-statistics	86.32963^***^	22.31128^***^	44.03888^***^	91.45425^***^

*** *significance level is at 0.01;*

* *is 0.1 significance level (Cross-section weights and white cross-section variance method)*.

Thus, in the first quarter of the year of 2020, with the severe effects of the COVID-19 outbreak, Chinese firms' revenues growth rates decreased by 39.94%, whereas their ROA and ROE dropped slightly by 0.3 and 0.8%, respectively, and other variables controlled. This outbreak reduced Chinese firms' ATO ratios by 5.2%, assuming no change in other variables. In other words, the COVID-19 explosion negatively influenced Chinese listed firms' financial performance in the quarter ended March 31, 2020. Besides the negative impacts of the COVID-19 attack on Chinese listed firms' financial performance, other determinants of their financial performance figure out in four regression models. In model (1), Chinese firms' liquidity and capital structure negatively affect their REGs, although such effects were modest (−0.01 and −0.075, respectively). In the COVID-19 outbreak context, firms with one-unit higher current ratio and one-unit higher financial leverage experienced 1 and 7.5% lower REGs when the remaining variables control. The industry-specific factors, CC, and CSR positively influenced Chinese listed firms' REGs with a significant coefficient at 0.01 levels. Furthermore, Chinese firms' ROA varied across industries, but this variation was modest because the coefficient of the industry variable was small (0.0004).

#### Effects of COVID-19 Outbreak on the Financial Performance

Table 5 provides outcomes of tests for the industry heterogeneity in impacts of the Covid-19 outbreak on Chinese firms' financial performance. The coefficient of the Medical industry was significant and positive in all models, so in the context of this outbreak, Chinese medical firms obtained higher financial performance, which is similar to the results of Sun et al. (56).

**Table 5:** Generalized linear regression with medicine and ATE industry.

**Dependent variable**	**REG**	**ROA**	**ROE**	**ATO**
C	0.004821	−0.013052	−0.066109	0.002817
COVID-19	−0.009374^***^	−0.005529^***^	−0.004138^***^	−0.005244^***^
Medicine	0.001352^***^	0.000511^***^	0.001258^***^	0.001169^***^
COVID-19^*^Medicine	0.004662^***^	0.001493^***^	0.001152^***^	0.001519^***^
ATE	−0.023942^***^	0.001449^***^	0.003028^***^	0.002685^***^
COVID-19^*^ATE	−0.014384^***^	−0.009186^***^	−0.000229^***^	−0.002356^***^
CC	0.003284^***^	0.002458^***^	0.002173^***^	0.001592^***^
CSR	0.001092^**^	0.0021817^**^	0.001302^**^	0.008309^**^
Liquidity	−0.002319	0.001173^*^	0.001083^*^	−0.001358
CCC	0.000013^*^	0.0000002^*^	0.000001^*^	−0.000036^*^
Capital structure	−0.062169	−0.002109^*^	−0.001752^*^	−0.252419
State ownership	−0.001056^*^	0.00000021^*^	0.0000012^*^	0.000107
Size	0.001263	0.001018^*^	0.001253^*^	0.000789
*F*-statistics	78.24398^***^	19.31872^***^	52.14027^***^	87.28372^***^

*** *is 0.01 significance level;*

** *is 0.05 significance level;*

* *is 0.1 significance level (cross-section weights and white cross-section covariance method)*.

By contrast, the coefficient of the ATE variable was significant and negative, so Chinese travel, entertainment, and airline firms obtained lower financial performance than others in this outbreak. The interaction terms between the Covid-19 attack and medicine were significant and positive in all models, the harmful effects of this outbreak on firms' financial performance in the medical industry reduce (56). Differently, the interaction terms between this outbreak and ATE were significantly negative in all models, It confirms that Hypothesis 2. Table 6 provides outcomes of the tests for the regional heterogeneity in impacts of the Covid-19 outbreak on Chinese listed firms' financial performance. The coefficients of DSR in all models were significant and negative, meaning that the Covid-19 attack negatively reduced the financial performance of firms located in the dangerously severe risky regions, although such adverse effects were modest.

**Table 6:** Generalized linear regression in serious regions.

**Dependent variables**	**REG**	**ROA**	**ROE**	**ATO**
C	0.002923	−0.082392	−0.012693	−0.001927
COVID-19	−0.004335^***^	−0.003532^***^	−0.002245^***^	−0.003544^***^
DSR	−0.001432^**^	−0.001930^**^	−0.002804^**^	−0.001082^**^
COVID-19^*^DSR	−0.003193^***^	−0.002291^***^	−0.002192^***^	−0.004138^***^
CC	0.002720^***^	0.001933^***^	0.001381^***^	0.001318^***^
CSR	0.001752^**^	0.001429^**^	0.001093^**^	0.001029^**^
Liquidity	−0.00226	0.001072^*^	0.001108^*^	−0.003025
CCC	−0.000902^*^	−0.004072	0.000003	−0.000712^*^
Capital structure	−0.001933	−0.001651^*^	−0.001523^*^	−0.000492
State ownership	−0.001923^*^	0.0000023	0.0000013	0.00000028
Size	0.002837	0.000184^*^	0.001224^*^	0.003717
*F*-statistics	96.44512^***^	35.82471^***^	47.90162^***^	95.24629^***^

*** *is 0.01 at significance level;*

** *is 0.05 at significance level;*

* *is 0.1 at significance level (cross-section weights and white cross-section covariance method)*.

Moreover, Table 7 provides the outcomes of tests for the heterogeneous impacts of the Covid-19 outbreak on Chinese firms' financial performance across DHR. The interaction terms between the Covid-19 attack and DHR were significant and negative in all models, so Chinese firms experienced the more severely damaging impacts of the COVID-19 outbreak than others in DHRs.

**Table 7:** Generalized linear regression in high-risky regions.

**Dependent variable**	**REG**	**ROA**	**ROE**	**ATO**
C	0.001038	0.018164	−0.012264^*^	−0.0031527
COVID-19	−0.045867^***^	−0.006243^***^	−0.072545^***^	−0.005436^***^
DHR	−0.008682^**^	−0.004853^**^	−0.004421^**^	−0.001488^**^
COVID-19^*^DHR	−0.005859^***^	0.004915^***^	0.004926^***^	0.005387^***^
CC	0.003120^***^	0.002384^***^	0.002819^***^	0.002072^***^
CSR	0.001942^*^	0.001209^*^	0.002247^*^	0.001529^*^
Liquidity	−0.00193	0.002742^*^	0.000450^*^	−0.000556
CCC	−0.001134^*^	−0.001787	−0.002452	−0.001249^*^
Capital structure	−0.000133	−0.000329^*^	−0.002246^*^	−0.001397
State ownership	−0.002019^*^	−0.001292	0.000329	0.002417
Size	0.001324	0.000412^*^	0.000412^*^	0.000513
*F*-statistics	97.43421^***^	39.98565^***^	58.26957^***^	95.67289^***^

*** *is 0.01 significance level;*

** *is 0.05 significance level;*

* *is 0.1 significance level (Cross-section weights and white cross-section covariance method)*.

The coefficient of interaction terms between the Covid-19 attack and DHR are stronger and the death cases of COVID-19 in different provinces and cities have substantial adverse effects on the financial performance of Chinese listed companies. Furthermore, Table 8 provides outcomes of tests for impacts of the Covid-19 outbreak on Chinese firms' financial performance across regions in China. The interaction terms between the Covid-19 attack and each area were negative and significant in all models. These terms were most important for the central region, so Chinese listed firms in the mid regions experienced the more substantial negative impacts of the COVID-19 outbreak on their financial performance than others. Compared with east and west, Hubei, an essential province in Central China, has been severely impacted by the attack. Overall, Hypothesis 3 is confirmed.

**Table 8:** Generalized linear regression in Chinese different zones.

**Dependent Variable**	**REG**	**ROA**	**ROE**	**ATO**
C	0.002453	0.002454	0.004549	0.001782
COVID-19	−0.003428^***^	−0.002143^***^	−0.001334^***^	−0.002446^***^
Coast	−0.001342^*^	−0.001729^*^	−0.001533^*^	−0.001619^*^
COVID-19^*^East	−0.002817^*^	−0.003569^*^	−0.002926^*^	−0.002871^*^
Mid	−0.000938^**^	−0.000193^**^	−0.00184^**^	−0.000748^**^
COVID-19^*^Central	−0.002342^**^	−0.001239^**^	−0.001286^**^	−0.002394^**^
West	−0.000221^*^	−0.000185^*^	−0.000153^*^	−0.000169^*^
COVID-19^*^West	−0.001329^*^	−0.000928^*^	−0.000712^*^	−0.001895^*^
CC	0.001668^***^	0.001367^***^	0.001768^***^	0.001352^***^
CSR	0.000832^*^	0.000287^*^	0.000432^**^	0.000513^**^
Liquidity	−0.003228	0.001039^*^	0.001543^*^	−0.001457
CCC	−0.000352^*^	−0.00239	−0.001325	−0.000847^*^
Capital structure	−0.001029	−0.001028^*^	−0.001392^*^	−0.000869^*^
State ownership	−0.002329^*^	−0.001918	−0.001303	−0.001836
Size	0.001129	0.001348^*^	0.001980^*^	0.001762
*F*-statistics	128.39173^***^	61.29183^***^	69.42754^***^	96.30183^***^

*** *is 0.01 significance level;*

** *is 0.05 significance level;*

* *is 0.1 significance level (cross-section weights and white cross-section covariance method)*.

To explore the most potent effects on the central region in China, Table 9 provides outcomes of tests for impacts of the Covid-19 outbreak on firms' financial performance in 6 provinces around the Hubei province, where the outbreak emerged. The interaction terms between the Covid-19 outbreak and DHB were significant and negative, so Chinese listed firms' financial performance around the Hubei province were most severely affected by this outbreak than others.

**Table 9:** Generalized linear regression in Hubei effects.

**Dependent variable**	**REG**	**ROA**	**ROE**	**ATO**
C	0.001244	−0.001048	−0.002693	−0.001257
COVID-19	−0.004351^***^	−0.002538^***^	−0.002713^***^	−0.000144^***^
DHB	−0.000676^**^	−0.000530^**^	−0.000354^**^	−0.00472^**^
COVID-19^*^DHB	−0.005134^***^	−0.003976^***^	−0.002628^***^	−0.004751^***^
CC	0.000385^**^	0.000279^**^	0.000281^**^	0.000298^**^
CSR	0.000152^*^	0.000169^*^	0.000193^*^	0.0000129^*^
Liquidity	−0.001926	0.001123^*^	0.001186^*^	−0.001174
CCC	−0.001134^*^	−0.001422	−0.002242	−0.001327^*^
Capital structure	−0.002681	−0.001917^*^	−0.001638^*^	−0.002438
State ownership	−0.000334^*^	0.00223	0.000192	0.000539
Size	0.002431	0.001354^*^	0.000847^*^	0.001182
*F*-statistics	99.83116^***^	58.64263^***^	53.03872^***^	97.34183^***^

*** *is 0.01 significance level;*

** *is 0.05 significance level;*

* *is 0.1 significance level (cross-section weights and white cross-section covariance method)*.

#### Moderating Effects of Corporate Social Responsibility

Table 10 provides outcomes of tests for the moderating role of CC and CSR for the relationship of Covid-19 outbreak and Chinese firms' financial performance. In four models, the interaction terms the Covid-19 attack and CC. As the moderating role of CSR for Covid-19 attack and CSR is significant and positive, so it confirms the Hypothesis 5–6. Corporate culture and CSR reduced the negative impacts of the COVID-19 attack on Chinese firms' financial performance. However, such moderating effects were modest (coefficients of the interaction terms were extremely small in four models).

**Table 10:** Generalized linear regression with CR and CSR.

**Dependent variable**	**REG**	**ROA**	**ROE**	**ATO**
C	0.001426	−0.001048	−0.001423	−0.001374
COVID-19	−0.002314^***^	−0.001258^***^	−0.001353^***^	−0.001685^***^
CC	0.002382^***^	0.001349^***^	0.001831^***^	0.001991^***^
COVID-19^*^CC	0.000120^***^	0.000096^***^	0.000104^***^	0.000112^***^
CSR	0.001228^**^	0.001029^**^	0.001038^*^	0.0000129^*^
COVID-19^*^CSR	0.000082^*^	0.000043^*^	0.000035^*^	0.000057^*^
COVID-19^*^Medicine^*^CC	0.008230^***^	0.005282^***^	0.004982^***^	0.005872^***^
COVID-19^*^ATE^*^CC	0.006831^**^	0.004338^**^	0.003281^**^	0.004482^**^
COVID-19^*^DSR^*^CC	−0.000827^**^	−0.000429^**^	−0.000381^**^	−0.000608^**^
COVID-19^*^DHR^*^CC	−0.001182^*^	−0.001093^*^	−0.000812^*^	−0.0001042^*^
COVID-19^*^East^*^CC	−0.000128^*^	−0.000127^*^	−0.000138^*^	−0.000192^*^
COVID-19^*^Central^*^CC	−0.003621^**^	−0.001823^**^	−0.001338^**^	−0.002142^**^
COVID-19^*^West^*^CC	−0.000372^*^	−0.000239^*^	−0.000216^*^	−0.000182^*^
COVID-19^*^DHB^*^CC	0.000225^*^	0.000142^*^	0.000129^*^	0.000231^*^
COVID-19^*^Medicine^*^CSR	0.002982^**^	0.002392^**^	0.002426^**^	0.001093^**^
COVID-19^*^ATE^*^CSR	0.002938^*^	0.002178^*^	0.001982^*^	0.000128^*^
COVID-19^*^DSR^*^CSR	−0.000292^*^	−0.000224^*^	−0.000183^*^	−0.000198^*^
COVID-19^*^DHR^*^CSR	−0.000394^*^	−0.000242^*^	−0.000221^*^	−0.000128^*^
COVID-19^*^East^*^CSR	−0.000117^*^	−0.000087^*^	−0.000079^*^	−0.000126^*^
COVID-19^*^Central^*^CSR	−0.000382^**^	−0.000212^*^	−0.000192^*^	−0.000223^**^
COVID-19^*^West^*^CSR	−0.000115^*^	−0.000098^*^	−0.000079^*^	−0.000143^*^
COVID-19^*^DHB^*^CSR	0.000213^*^	0.000168^*^	0.000192^*^	0.000179^*^
Liquidity	−0.000382	0.000283^*^	0.000155^*^	−0.002564
CCC	−0.000313^*^	−0.002556	−0.001426	−0.000455^*^
Capital structure	−0.002861	−0.000496^*^	−0.000835^*^	−0.002863
State ownership	−0.000230^*^	0.000289	0.000232	0.000736
Size	0.000134	0.000692^*^	0.000324^*^	0.000122
*F*-statistics	129.82463^***^	82.98143^***^	86.82736^***^	117.98133^***^

*** *is 0.01 significance level;*

** *is 0.05 significance level;*

* *is 0.1 significance level (cross-section weights and white cross-section variance method)*.

We test the interactions COVID-19 outbreak, the medicine industries, and the CC test. These interaction terms were significant and positive, so Chinese medical firms with good CC could reduce the negative impacts of the Covid-19 outbreak on their financial performance. For ATE industry interaction terms between this industry, the Covid-19 attack and CC added, coefficients of these terms were significant and positive so that CC could reduce the negative impacts of this outbreak. Similarly, CSR reduces the harmful effects of the COVID-19 spell on the financial performance of both the medicine and ATE. Moreover, CC is significant and negatively influences the relationship between the Covid-19 outbreak and firms' financial performance in serious regions. However, the moderating role of CSR for the negative relationship between the Covid-19 outbreak and firms' financial performance in this region confirms. To the high risky regions, the interaction terms between the Covid-19 attack, DHR, and CC and the interaction terms between this outbreak, DHR, and CSR were significant and negative in all models. Table 11 provides outcomes of tests for the moderating role of Chinese state-owned listed companies' CC and CSR for the relationship between the Covid-19 outbreak and financial performance. The state ownership moderated the relationship between the COVID-19 outbreak and Chinese firms' financial performance. The SOEs experienced fewer negative effects of the COVID-19 outbreak on their financial performance than non-SOEs. Regarding the moderating effects of CC and CSR, the interaction terms between these variables, the COVID-19 outbreak, and DSO were positive and significant in all models.

**Table 11:** Generalized linear regression in state-ownership list companies.

**Dependent variable**	**REG**	**ROA**	**ROE**	**ATO**
C	0.027817	−0.022708^***^	−0.056606^***^	0.108270^***^
COVID-19	−0.003135^***^	−0.002306^***^	−0.002418^***^	−0.003216^***^
DSO	0.000343^***^	0.000221^***^	0.000127^***^	0.000259^***^
COVID-19^*^DSO	0.001431^***^	0.001325^***^	0.001164^***^	0.001273^***^
CC	0.000832^***^	0.000563^***^	0.000329^***^	0.000664^***^
COVID-19^*^DSO^*^CC	0.000272^*^	0.000118^*^	0.000105^*^	0.000198^*^
CSR	0.000629^**^	0.000483^**^	0.000276^**^	0.000489^**^
COVID-19^*^DSO^*^CSR	0.000517^*^	0.000386^*^	0.000281^*^	0.000392^*^
Liquidity	−0.001323	0.001124^*^	0.001273^*^	−0.009049
CCC	−9.05E-05^*^	−4.04E-07	2.48E-07	−2.78E-06^*^
Capital structure	−0.084562	−0.001925^*^	−0.001341^*^	−0.25172
Size	0.002324	0.002461	0.005835	0.015371
*F*-statistics	155.7781^***^	21.30743^***^	46.73687^***^	72.72873^***^

*** *is 0.01 at significance level;*

** *is 0.05 at significance level;*

* *is 0.1 at significance level (cross-section weights and white cross-section covariance method)*.

## Conclusions and Recommendations

This study found that after controlling for firms' particular characteristics and industry-specific conditions, the COVID-19 epidemic harmed Chinese listed companies' financial efficiency, as their sales growth rate, ROA, ROE, and ATO decreased dramatically in the first quarter of 2020. As a result, the first hypothesis (H1) is accurate. However, aside from the significant impact on sales growth, the COVID-19 epidemic had only minor adverse effects on ROA, ROE, and ATO, with only 0.3, 0.08, and 5.2%, respectively. Hassan et al. (51) found that the effects of the COVID-19 epidemic on Chinese listed companies' financial results are not uniform, confirming the second hypothesis (H2). Furthermore, this study found that companies with a conservative working capital management approach could reduce COVID-19's adverse results. In terms of the moderating influence of CC and CSR, this study discovered that CC and CSR mitigated the negative effects of the Covid-19 epidemic on Chinese firms' financial results, confirming the H4 and H5 hypotheses. The H6 supports this study, which found that state ownership moderated the relationship between the COVID-19 and economic consequences of Chinese listed companies. Since the relationship terms between the COVID-19 outbreak and state ownership is favorable, state ownership minimized the severity of the outbreak's negative impact on the financial output of Chinese listed companies. In other words, SOEs' sales growth rate, ROAs, ROE, and stock turnover was less affected by the epidemic than others. The ability to generate revenue from the Travel and Leisure, Retailer, Healthcare, and Basic Materials industries was highest in ATO, while the ability to generate revenue from assets of Technology, Paper manufacturing, and other sectors was lowest.

## Suggestions for Future Research

Despite the accomplishments of this study and its essential contribution to the literature, it nevertheless has two significant flaws. The results of the study point to several practical consequences for clinicians related to improvements in market practices. Firms should use a cautious working capital management approach rather than an offensive working capital management strategy in the event of a viral epidemic. In the light of supply chain delays triggered by the health crisis, the traditional working capital management approach also works.

## Data Availability Statement

The raw data supporting the conclusions of this article will be made available by the authors, without undue reservation.

## Author Contributions

YS: data analysis, writing, and supervision. YL: writing, discussion, and analysis. All authors contributed to the article and approved the submitted version.

## Conflict of Interest

The authors declare that the research was conducted in the absence of any commercial or financial relationships that could be construed as a potential conflict of interest.

## Publisher's Note

All claims expressed in this article are solely those of the authors and do not necessarily represent those of their affiliated organizations, or those of the publisher, the editors and the reviewers. Any product that may be evaluated in this article, or claim that may be made by its manufacturer, is not guaranteed or endorsed by the publisher.
